# Glow Fish: A New Biosensor to Detect How Environmental Estrogens Affect Tissues

**DOI:** 10.1289/ehp.120-a284b

**Published:** 2012-07-02

**Authors:** Wendee Holtcamp

**Affiliations:** Wendee Holtcamp, based in Houston, TX, has written for *Nature*, *Scientific American*, *National Wildlife*, and other magazines.

Chemicals that mimic natural estrogens have well-documented effects on the reproductive systems of vertebrates, typically acting as endocrine disruptors, but scientists know much less about how these chemicals affect other tissues and body systems. Investigators have explored zebrafish transgenically modified to glow with green fluorescent protein (GFP) as a way to detect effects of different estrogenic chemicals in real time. A British team now confirms and improves upon previous work by developing an even more sensitive transgenic zebrafish biosensor for assessing potential endocrine disruptors [*EHP* 120(7):990–996; Lee et al.].

Earlier transgenic models reported exposure only in certain tissues or at very high doses. In the current study, the investigators exposed embryonic transgenic zebrafish at 1 hour postfertilization to environmentally relevant doses of five different estrogenic chemicals: the plasticizer bisphenol A (BPA), the industrial surfactant 4-nonyl-phenol (NP), the natural steroids estrogen and 17β-estradiol (E_2_), and 17α-ethinylestradiol (EE_2_), a synthetic hormone widely used in contraceptive pills and hormone replacement therapy.

**Figure:**
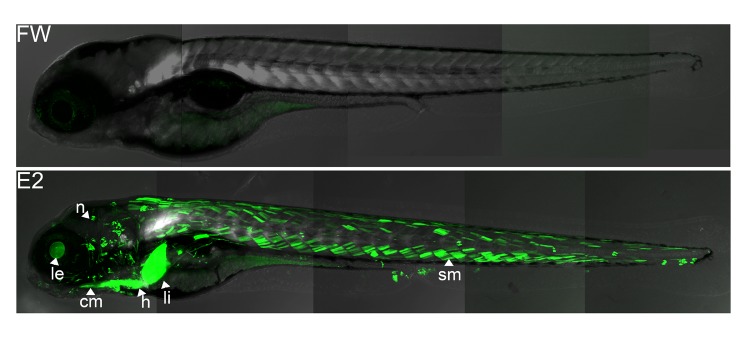
A new transgenic zebrafish model offers a more sensitive method for studying responses to environmentally relevant doses of estrogenic chemicals. These images show 4-day-old zebrafish larvae exposed as embryos to water alone (top) or to water spiked with 100 ng/L E2 (bottom). Tissues affected by the estrogen exposure glow green—in this case, the cranial muscle (cm), heart (h), lens (le), liver (li), neuromasts (n) of the lateral line, and somite muscle (sm). Lee et al., doi: 10.1289/ehp.1104433

Tissues such as the liver, forebrain, lateral line (a sensory system found in aquatic vertebrates), otic vesicles, eyes, heart, and cranial and somite muscle appeared to be strongly affected by some of the compounds tested, but not others. The study also found that different tissues’ response to EE_2_ was time dependent. For example, liver, heart, and muscle tissue began to glow 24 hours postfertilization and reached maximum strength at 96 hours, whereas a significant increase in fluorescence was seen in the otic vesicles 48 hours postfertilization, and in the eyes and forebrain after 72 hours.

Based on this study, the zebrafish biosensor seems a promising and powerful system to test the impacts of a variety of estrogenic chemicals in a living vertebrate at low, environmentally relevant doses. The authors suggest the system could be developed for high-throughput screening, an urgent need in the study of endocrine-disrupting chemicals.

